# Genetic structure of Australian glass shrimp, *Paratya australiensis,* in relation to altitude

**DOI:** 10.7717/peerj.8139

**Published:** 2020-01-09

**Authors:** Sharmeen Rahman, Daniel Schmidt, Jane M. Hughes

**Affiliations:** Griffith School of Environment and Australian Rivers Institute, Griffith University, Brisbane, QLD, Australia

**Keywords:** Local adaptation, Genetic structure, Stream network, Freshwater shrimp, Outliers

## Abstract

*Paratya australiensis* Kemp (Decapoda: Atyidae) is a widely distributed freshwater shrimp in eastern Australia. The species has been considered as an important stream organism for studying genetics, dispersal, biology, behaviour and evolution in atyids and is a major food source for stream dwelling fishes. *Paratya australiensis* is a cryptic species complex consisting of nine highly divergent mitochondrial DNA lineages. Previous studies in southeast Queensland showed that “lineage 4” favours upstream sites at higher altitudes, with cooler water temperatures. This study aims to identify putative selection and population structure between high elevation and low elevation populations of this lineage at relatively small spatial scales. Sample localities were selected from three streams: Booloumba Creek, Broken Bridge Creek and Obi Obi Creek in the Conondale Range, southeast Queensland. Six sample localities, consisting of 142 individuals in total were sequenced using double digest Restriction Site Associated DNA-sequencing (ddRAD-seq) technique. Among the 142 individuals, 131 individuals shared 213 loci. Outlier analysis on 213 loci showed that 27 loci were putatively under selection between high elevation and low elevation populations. Outlier analysis on individual streams was also done to test for parallel patterns of adaptation, but there was no evidence of a parallel pattern. Population structure was observed using both the 27 outliers and 186 neutral loci and revealed similar population structure in both cases. Therefore, we cannot differentiate between selection and drift here. The highest genetic differentiation was observed between high elevation and low elevation populations of Booloumba Creek, with small levels of differentiation in the other two streams.

## Introduction

Genetic variation within and among populations arises as a result of a number of factors, namely-mutation, genetic drift, gene flow/dispersal and natural selection (Slatkin, 1985). Widespread gene flow homogenizes genetic variation among populations whereas restricted gene flow creates genetic divergence among populations due to genetic drift and/or natural selection (Slatkin, 1985; Hughes, Schmidt & Finn, 2009). In a dendritic stream network, geneflow and population connectivity may be influenced by the stream’s physical features resulting in population structuring within and between subcatchments within a larger stream catchment (Meffe & Vrijenhoek, 1988; Hughes, Schmidt & Finn, 2009). Additionally, altitudinal differences (high elevation and low elevation) are very useful for exploring evolutionary adaptation over short distances because changes in environmental conditions occur across transects and organisms may be adapted to the local environment (Körner, 2007).

Population genomic investigations are powerful approaches for disentangling stochastic and deterministic processes generating population genetic subdivision across space and habitat. When studying population genomics, it is important to consider both neutral loci and loci under selection in order to illustrate population demography clearly. Neutral loci will give information on population demography and evolutionary history whereas loci under selection can show evidence of adaptation (Ellegren & Sheldon, 2008). Population structure is usually assessed using neutral loci, although loci under selection should also be identified as they represent an important component of robust demographic inference about the evolutionary processes that are structuring the populations. Loci under selection (outliers) can show extreme variation and it is wise to use both neutral and loci under selection separately to understand the actual population structure (Andr’e et al., 2011).

Restriction site Associated DNA-sequencing (RAD-seq) is a technique where thousands of genetic markers (SNP’s) can be identified across the genome (Davey & Blaxter, 2011). Compared to other markers (microsatellites, AFLPs and RFLPs), which require prior information of the species of concern, the RAD-seq technique can be applied to an organism without a reference genome producing SNP markers in non-model organisms very efficiently (Helyar et al., 2011; Mastretta-Yanes et al., 2015). Hence, both neutral and non-neutral SNP markers are produced through RAD-seq, thus providing possibilities for studying both population demography and natural selection in species.

**Figure 1:**
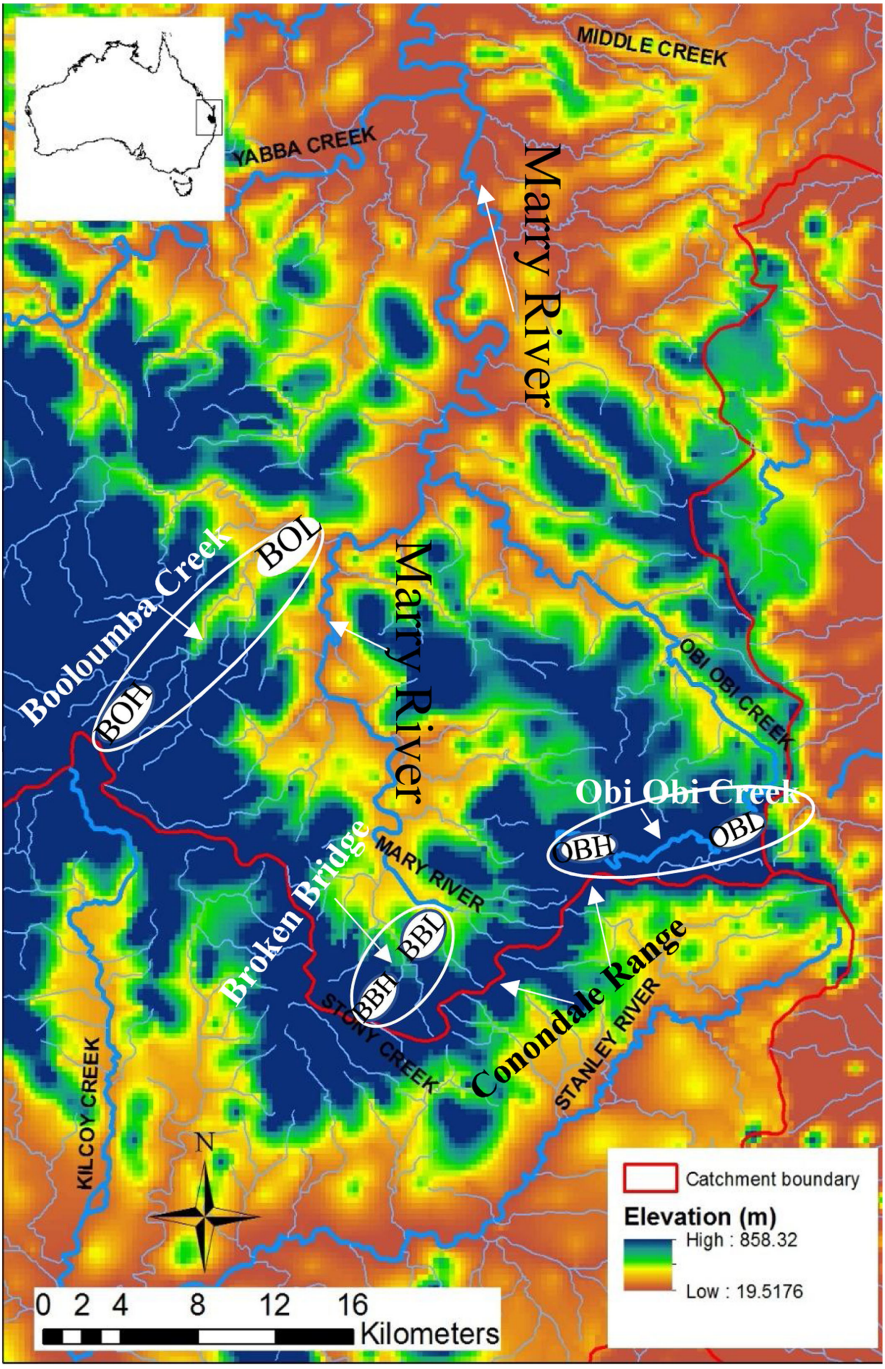
Map of the Conondale Range showing sampling sites. Blue colours in the map indicate high altitude sites while red-yellow indicates low altitude sites. BOH, Booloumba High site; BOL, Booloumba Low site; BBH, Broken Bridge High site; BBL, Broken Bridge Low site; OBH, Obi Obi High site; OBL, Obi Obi Low site.

The Australian glass shrimp, *Paratya australiensis* is a small bodied (2–4 cm long) freshwater shrimp. Additionally, there are some estuarine populations in southern Victoria. The shrimp has a filter feeding and scavenging habit and plays a key role in lotic community structure (Moulton et al., 2012). Freshwater fishes like, *Mogurnda adspersa*, and *Tandanus tandanus* feed largely upon *P*. *australiensis* in the lower reaches of rainforest streams (Williams, 1977). *P*. *autraliensis* has a distribution ranging from south-eastern South Australia including Tasmania to south- western New South Wales and extends northwards to the Atherton Tablelands on the eastern coast of Queensland (Bishop, 1967; Williams, 1977). Although in southern Australia *P*. *australiensis* is not abundant in upland streams (Williams, 1977), they are obviously the most abundant macroinvertebrate in the small pools of upland subtropical rainforest streams in southeast Queensland, especially in the Conondale Range; this part of the water-shed separates two large river systems: the Brisbane River to the south and the Mary River to the north, (Fig. 1). Breeding of *Paratya* occurs from the end of spring to early summer when the water temperature is higher. Planktonic larvae appear late in the dry season (October) and are sometimes present through to February. Late breeding and larval development is observed at the high-altitude sites, which suggests the importance of temperature to this species (Hancock & Bunn, 1997). Eggs and early stage larvae vary in size between freshwater and estuarine environments, with larger eggs and larvae in the freshwater environment (Walsh, 1993). *Paratya* has been considered to have a high dispersal ability due to its wide distribution in upland and lowland streams and freshwater throughout the east coast of Australia (Cook et al., 2006). However, several more recent studies have found restricted dispersal in this species (Hughes et al., 1995; Hurwood et al., 2003). Although there is still only a single species described, recent studies have shown that there are nine highly divergent COI mtDNA lineages of *P*. *australieneis* and that it probably represents a cryptic species complex. Some of these lineages are geographically restricted and others are widely distributed across temperature and elevational gradients along eastern Australia (Cook et al., 2006). Further, some of these lineages have been found to coexist (Cook, Bunn & Hughes, 2007; Baker et al., 2004). Among the nine highly divergent lineages, lineage 4, 6 and 8 are most abundant (Cook et al., 2006). The divergence between the cryptic species appear to have arisen due to a number of amphidromy-freshwater life history transitions rather than historical isolation of amphidromous populations (Cook et al., 2006).

Although cryptic lineages were identified in 2006 (Cook et al., 2006), in 1993, it was still considered a single species, and to study the instream movement pattern of *P*. *australiensis*, there was a translocation undertaken. The translocation was done in the Brisbane River catchment in the Conondale Range, from Kilcoy Creek to Branch Creek (Hancock & Hughes, 1999). It was later determined that the two creeks contained two different lineages of *Paratya*. Kilcoy Creek, which is at a higher elevation (∼520 m asl) contained lineage 4, while Branch Creek, which is at a lower elevation (∼400 m asl), contained lineage 6 (Hurwood et al., 2003). These lineages diverged from each other approximately 2.3 million years ago (Hurwood et al., 2003) so the translocation event created a “secondary contact zone” between them (Wright, 2012). An early study showed that some hybridization between the lineages had occurred but that lineage 4 had dispersed more than 1.5 km upstream of the translocation site and had sent lineage 6 almost extinct in all sites higher and 2 sites lower of than the original translocation site (Hughes et al., 2003). Two years later, lineage 4 sill dominated high elevation sites (In the Branch Creek-translocated stream) but had not moved further to low elevation sites (Fawcett, Hurwood & Hughes, 2010). It was hypothesized that lineage 4 is adapted to higher elevations, as they quickly colonized the upper reaches of the stream which is approx. 1–2 °C cooler than the lower elevation sites. This was supported by a small laboratory experiment in which individuals of the 2 lineages were incubated in separate tanks and water temperature was increased by 1 °C every 3 days. Lineage 4 was less tolerant to increasing temperature than lineage 6, dying at a lower temperature (Fawcett, Hurwood & Hughes, 2010). Ten years later, in Branch Creek following very heavy rainfall over 2 summers, lineage 4 had shifted further to downstream, which was suspected to be due to lower temperatures caused by heavy rainfall (Wilson, Schmidt & Hughes, 2016), favoring lineage 4 over lineage 6. Given that temperature appears to be an important environmental factor for the distribution of *Paratya australiensis* lineages in previous studies, we chose to examine evidence within a single lineage, Lineage 4, for adaptation to different altitudes in a different catchment, the Mary River Catchment. This catchment was chosen as it had been shown to contain only a single lineage.

The concept of restricted dispersal of *P*. *australiensis* in the Conondale Range was reported in 1995 using allozyme analysis (Hughes et al., 1995). The study included 15 sites in total with 3 headwater stream sites and a confluence site in each of four subcatchments. The subcatchments were Booloumba and Mary subcatchments in the Mary River catchment and Kilcoy and Stony subcatchments in the Brisbane River catchment area. The study reported that upstream populations from Booloumba Creek and Kilcoy creek were significantly different from populations at the stream confluence sites, suggesting limited dispersal within a stream (Hughes et al., 1995). Furthermore, in another study by Hurwood et al. (2003), population structure of *P*. *australienesis* in the Conondale range reflected contemporary restricted gene flow (with allozyme data) but mtDNA COI data suggested that there was no contemporary dispersal between up and downstream populations. So, there is a need for a larger data set to observe population structure in the Conondale Range even at this small spatial scale.

The aims of the present study were to investigate population structure at a small spatial scale using genomic data and determine if loci putatively under selection between high elevation and low elevation populations indicate evidence of adaptation to altitude. We were interested to determine if the divergence between populations is influenced by adaptation and selection or genetic drift. Adaptation and selection can act on much shorter timescales than genetic drift to drive divergence of populations. Thus, loci under strong selection may reveal population genetic structure between populations from different ecological habitats neutrally evolving loci may not. This pattern would provide clear evidence that selection/adaptation is working to structure these populations. If the same pattern of population genetic structure is observed in both loci under selection and neutrally evolving loci, there is little evidence of selection acting on these populations. Drift and selection are not mutually exclusive processes, and divergence that may have been initially driven by selection can also result in the same pattern via genetic drift if dispersal is also limited in the system. For example, a chance dispersal event to a new habitat followed by local adaptation and complete isolation from the parental population will show evidence of selection early on in the divergence process and also show evidence of neutral genetic drift following an extended period of isolation. In order to achieve our goals sampling was undertaken from both high and low altitude populations from each of three creeks belonging to a stream network. Outlier analysis was undertaken to detect loci putatively under selection, and later both neutral and adaptative loci were used to examine population structure.

## Materials & Methods

### Sampling

The sampling sites were in the Conondale Range which extends from 26°55′S, 152°45′E northwest to 26°37′S, 152°30′E in south-east Queensland and is 100 km northwest of Brisbane. The Conondale Range occupies the area which separates two large river systems, the Brisbane River to the south and the Mary River to the north (Hughes et al., 1995). As this region is subtropical rainforest, it has cool dry winters and hot wet summers with a mean annual rainfall of approximately 1134.7 mm (Australian Bureau of Meteorology, 2015). The upland area is mostly rainforest (>500 m asl). Headwater streams are prevalent on either side of the range and the streams flow into the major river networks, the Brisbane River and the Mary River (Hughes et al., 1995).

Three subcatchments Booloumba Creek (BOH = 26°41.062S, 152°37.185E, BOL = 26°37.960S, 152°39.124E), Broken Bridge Creek (BBH = 26°50.483S, 152°44.276E, BBL = 26°47.59S, 152°42.399E) and Obi Obi Creek (OBH = 26°46.254S, 152°48.644E, OBL = 26° 75.92S, 152°87.09E) (Fig. 1) were selected, all of which fall in the greater Mary River catchment Area. High elevation and low elevation sites were ∼50 km away from each other. Sites were selected in the headwater (upstream) and in the downstream locations from each of these streams. There are waterfalls (>10 m) in Booloumba Creek and in the Obi Obi Creek upstream area. Upstream to downstream site temperatures differ by 1–3 °C (Mosisch & Bunn, 1997).

Individuals from the Booloumba Creek High site are denoted as BOH, Booloumba Creek Low site as BOL, Broken Bridge High site are as BBH, Broken Bridge Low site as BBL and Obi Obi High as OBH and Obi Obi Low site as OBL. High altitude sites were at ∼550 m asl and low altitude sites were ∼100 m asl.

*Paratya australiensis* were collected from 2014–2016. Shrimps were collected from small pools at each site using a seine net and a dip net. 25–35 individuals were collected from each site (Table S1). As the RAD-library preparation requires very high-quality DNA, the shrimps were preserved immediately in 100% ethanol and kept at −20 °C in the laboratory.

### Molecular methods

DNA was extracted from the abdominal tissue (25 mg) of each individual using DNA-easy Blood and Tissue kits (QIAGEN, GmbH, D-40724, Hilden, Germany) following the manufacturer’s instructions. DNA was eluted to 50 µl final volume. 4 µl of RNase-A (10 mg/ml) was added to the extraction and incubated at room temperature for 10 min. DNA concentration was quantified using a Qubit 3.0 fluorometer from Thermo Fisher Scientific according to the kit instructions. Samples containing 1.5 ng of DNA were used for ddRAD library.

A double digest RAD library (ddRAD) was prepared at the Australian Genomic Research Facility (AGRF), Melbourne, Australia. The restriction enzyme combination used for this library preparation were PstI/HypCH4IV. Five types of barcodes were used ranging from 4 bp to 8 bp in size. ddRAD libraries prepared from the 142 individuals from high elevation and low elevation populations of all 3 streams were sequenced using Illumina NextSeq500 platform of the Australian Genomic Research Facility (AGRF). Single end sequencing was done in High output mode for 150 cycles.

### SNP filtering

Preliminary analysis of the FASTQ reads was undertaken with the programme Stacks v.1.35 (Catchen et al., 2013). All sequences were trimmed to 140bp with Phred quality score of +33. During demultiplexing, no mismatches were allowed in barcodes. Afterwards, all reads were used for a *de novo* assembly. The Stacks script *denovo*_*map*.*pl* was run to assemble the reads into stacks, build a tag catalogue and match individual samples to the catalogue. For the *denovo*_*map*.*pl*, the assembly parameters set were: minimum coverage value of -m 3; minimum number of mismatches allowed between loci while processing a single individual was -M 2; minimum number of mismatches allowed between loci when building the catalogue was -n 1. Next, the Populations program was run in Stacks v.1.35 to output sample genotype data and SNPs into different common formats for population genetics programs. The Populations program runs using the processed outputs of the *denovo*_*map*. The following filtering parameters were used: loci present in all 6 populations -p 6, loci present in 75% of the individuals in each population (*r* = 0.75), a minimum depth of coverage for each individual of -m 10. A minor allele frequency of 5% (-min_maf 0.05) was also applied to exclude loci with minor allele frequency of less than 5%. A whitelist was made with loci containing 2 haplotypes and then a single SNP per locus was selected from those haplotypes. This produced a white list of a single SNP per locus.

Genotypes were exported in different file formats including Structure, Fasta, vcf and Genepop format. vcf files generated from the Populations program were converted into the file formats necessary for downstream analyses using PGDspider v.2.0.5.0 (Lischer & Excoffier, 2011).

In the final output files, Broken Bridge High (BBH) had 17 individuals, Broken Bridge Low (BBL) had 29 individuals, Booloumba High (BOH) had 24, Booloumba Low (BOL) had 15, Obi Obi High (OBH) had 18 and Obi Obi Low (OBL) had 28 individuals. In total 131 individuals from 6 populations were used for subsequent analysis. 213 loci, each containing a single SNP in 131 individuals from 6 populations, were used for estimating genetic diversity, population structure and outlier analysis. Outlier analysis was also undertaken on each individual stream and compared to one another in order to identify any parallel pattern of adaptation.

### Statistical analysis

### Estimation of summary statistics

Summary statistics (number of individuals genotyped, number of private alleles, number of polymorphic sites, major allele frequency, observed heterozygosity, nucleotide diversity (pi), and Wright’s F statistics (*F*_IS_)) were calculated for RAD data using the populations pipeline of Stacks v.1.35 (Catchen et al., 2013).

Tests for conformance to Hardy–Weinberg Equilibrium (HWE) were done with Exact tests for each locus using default settings in Arlequin v.3.5 (Excoffier & Lischer, 2010). Sequential Bonferroni correction was applied to the *P* values. Also, pairwise *F*_ST_ estimates were calculated for all population pairs. Significance was assessed based on 1,000 permutations. The same version of Arlequin was used for this purpose.

### Estimating population structure

Two methods were employed here to identify population structure. Admixture analysis was used to detect genetic structure among all populations using the R package LEA (Frichot & François, 2015). Similar to Bayesian clustering programs like Structure (Pritchard, Stephens & Donnelly, 2000), LEA includes an R function to estimate individual admixture coefficients from small to very large genotypic matrices. For this study, as there were 6 populations, the probability of the admixture model was tested for clusters (K) ranging from 1-6, using 10 repetitions for each K value, 1 CPU was chosen to run the program and cross entropy was used to get the best number of ancestral populations.

Population structure analysis was also done using individual-based Principal Component Analysis (PCA) implemented in the R package Adegenet (Jombart, 2008). Here 213 loci were used for 131 individuals across the 6 populations. Later, PCA was done using the same number of loci, but excluding the two Booloumba Creek populations. In addition, PCA was also done excluding outliers and using only the loci presumed to be neutral.

### mtDNA barcoding and phylogenetic analysis of Booloumba Creek individuals

Due to the large divergence between the two populations from Booloumba Creek, 7 samples (3 samples from Booloumba Creek High site (BOH) and 4 samples from Booloumba creek low site (BOL) were sequenced for the mitochondrial COI gene. This was in order to confirm the mtDNA lineage to which they belonged. Details of the barcoding of Booloumba Creek individuals is described in Rahman, Schmidt & Hughes (2018).

### Detection of outliers

Bayescan v.2.1 (Foll & Gaggiotti, 2008) was used to detect *F*_ST_ outliers. An FDR of 0.001 was used. The logarithmic scale proposed by Jeffreys (1961) was used to determine the level of selection. 20 pilot runs with run length of 50,000 and an additional burn-in of 5000 were set. Prior odds were set to 10 for combined analysis including 6 populations and 213 loci. *F*_ST_ outlier analysis was also done using the program Arlequin v.3.5 (Excoffier & Lischer, 2010). The outlier detection method in Arlequin v.3.5 (Excoffier & Lischer, 2010) is based on the FDIST approach of Beaumont & Nichols (1996). This method is thought to produce a large number of false positives if the populations are distributed in a hierarchical manner, as is likely for populations in a stream network. Hence, a Hierarchical Island Model was implemented in Arlequin v.3.5 (Excoffier, Hofer & Foll, 2009). For (default) hierarchical analysis 20,000 simulations, 100 demes per group, 10 simulated groups were used. The 6 populations were grouped in 3 groups each containing an high elevation and a low elevation population. Loci that are on either tail of the distribution (<1% quantile and >99% quantile) are considered under selection. Loci above the 99% quantile are under divergent selection and loci below 1% quantile are under balancing selection.

The Pcadapt package was used to identify loci under selection based on Principal Component Analysis (PCA) method. After Principal Component 4 the scree plot reached a plateau so *K* = 4 was chosen which should include most of the variation. The package *q*-value (Storey & Bass, 2003) was used along with Pcadapt to convert *p*-values to qvalues (qvalue of 0.001 was used). *q* value is used when multiple testing is done whereas for single testing the *p* value is used. The *q* value is the minimum FDR at which a locus becomes significant (Foll & Gaggiotti, 2008).

### Summary statistics on the outliers

Genotype frequency of the outliers was observed using Adegenet in R. In order to observe deviation of any outlier locus from HWE (Hardy Weinberg Equilibrium) the Pegas package (Paradin, 2010) in R was used. Sequential Bonferroni Correction was applied to the *P* values. Furthermore, using the 213 loci, outlier analysis (*F*_ST_ outlier test, Bayesian and PCA based method) was performed on Booloumba populations only (BOH and BOL) and the outliers obtained were matched against the outliers obtained from the overall analysis. The analysis with only Booloumba Creek sites was done to observe if the outliers obtained in the overall analysis were much influenced by Booloumba Creek populations or not. If the outliers obtained in the overall analysis all matched with the outlier analysis on Booloumba Creek populations only, it would indicate high influence of Booloumba Creek population on the overall analysis and vice versa if there was less match.

### Parallel pattern of adaptation

In order to determine whether there were parallel patterns of adaptation between high elevation and low elevation populations each stream was analysed separately for evidence of outliers. The *F*_ST_ outlier test, the PCA-based outlier test and the Bayesian method were followed for each stream separately. Outliers detected in each stream population were listed in a tabular format and catalog ID of the outliers were compared to find common outliers across streams.

Furthermore, PCA was done based on the outliers produced in any of the 3 outlier detection methods using 27 loci (131 individuals) from all 6 populations. If there is a parallel pattern of adaptation across all three streams there should be one grouping of high elevation populations and another grouping for low elevation populations based on the outliers, due to similar pattern of divergence (Larson, 2015). The Adegenet Package in R (Jombart, 2008) was used for this purpose.

An additional PCA was done excluding all the outliers to observe the structuring of the populations and compare the results with the PCA including outliers.

## Results

### Sequencing, SNP discovery and filtration

We obtained RAD data from 142 Individuals. Lowest number of demultiplexed reads (40,991) were observed in the Obi Obi Creek samples and highest number of reads (21,338,955) were observed in Broken Bridge samples. Retained reads ranged from 7,438 (Obi Obi) to 21,321,266 (Broken Bridge).

*Denovo* assembly for 142 individuals produced 2,666,679 loci in the catalog and the depth of coverage ranged from 4.888x to 33.884x per individual. A white list was created with 207,619 loci which had only two haplotypes. Using these loci and selecting only a single SNP per locus generated 85,401 SNPs in 142 individuals.

Individuals with very low depth of coverage (below 10x) were excluded from the popmap, which resulted in 11 individuals being removed leaving 131 individuals in total. The Populations program was run on 85,401 loci, using filters described in methods. This generated 213 SNP’s in 131 individuals, common across all 6 populations. Once filtering was complete, the number of loci sequenced per individual varied from 4 to 213, with a mean of 109 loci per individual and a total of 213 variable loci overall.

### Genetic diversity

When only variant (only polymorphic nucleotide) positions were considered, the average number of individuals sampled at a locus ranged from 14.63 (BOL) to 27.17 (BBL). Only the Booloumba High population (BOH) had private alleles (25 alleles). Expected heterozygosity was more or less similar across all populations, ranging from 0.2159 to 0.2781 with the lowest in OBH and the highest in BBH. Nucleotide diversity ranged from 0.2231 (OBH) to 0.2877 (BBH) (Table 1). Upstream sites had higher nucleotide diversity than low sites for Broken Bridge and Booloumba Creek, whereas the OBL population had higher nucleotide diversity than OBH.

**Table 1:** Summary statistics for all 6 populations (Broken Bridge High-BBH, Broken Bridge Low-BBL, Booloumba High-BOH, Booloumba Low-BOL, Obi Obi High-OBH, Obi Obi Low-OBL) calculated for only polymorphic nucleotide positions (variant positions). Statistics are calculated per population and include the mean number of individuals genotyped per locus (N), the number of alleles that are unique to the group (Private), the average frequency of the major allele (P), the mean observed heterozygosity (H_obs_), the mean expected heterozygosity (H_exp_), the mean nucleotide diversity (pi- *π*), and the mean inbreeding coefficient (*F*_IS_).

	**N**	**Private**	**P**	**H**_**obs**_	**H**_**exp**_	*π*	*F*_**IS**_
**Variant Positions**							
BBH	15.1596	0	0.7891	0.2706	0.2781	0.2877	0.0572
BBL	27.1784	0	0.7968	0.3253	0.2701	0.2752	−0.1074
BOH	22.9531	25	0.8295	0.2100	0.2193	0.2242	0.0400
BOL	14.6338	0	0.8402	0.2082	0.2164	0.2241	0.0459
OBH	15.5070	0	0.8379	0.1948	0.2159	0.2231	0.0743
OBL	26.1690	0	0.7970	0.3312	0.2684	0.2737	−0.1324

*F*_IS_values were higher in upstream sites (BBH 0.0572, OBH 0.0743) than in downstream sites (BBL -0.1074, OBL -0.1324). In Broken Bridge and Obi Obi Creeks, individuals from downstream populations had an excess of heterozygotes as indicated by the negative *F*_IS_ values. Booloumba High and Low populations had similar *F*_IS_ values (Table 1).

Three loci deviated from Hardy Weinberg Equilibrium (HWE) but the deviation of these loci was not consistent across all populations. So, they were kept in the overall analysis. In the overall comparison, genetic differentiation (*F*_ST_ = 0.4461) was highly significant. The *F*_ST_ value between BOH and BOL (*F*_ST_ = 0.44616) was much higher than BBH and BBL (*F*_ST_ = 0.04289) although both were significant. OBH and OBL were not statistically differentiated from each other compared to the other population pairs (Table 2).

**Table 2:** Pairwise *F*_ST_ estimates among 6 populations of Paratya australiensis.

	**BBH**	**BBL**	**BOH**	**BOL**	**OBH**	**OBL**
**BBH**	0					
**BBL**	0.04289^***^	0				
**BOH**	0.35989^***^	0.43836^***^	0			
**BOL**	0.13869^***^	0.09618^***^	0.44616^***^	0		
**OBH**	0.07480^***^	0.00967	0.45632^***^	0.21252^***^	0	
**OBL**	0.04881^***^	−0.01681	0.44142^***^	0.10639^***^	0.00737	0

**Notes.**

*** *P* value <0.001.

BBH Broken Bridge High BBL Broken Bridge Low BOH Booloumba High BOL Booloumba Low OBH Obi-Obi High OBL Obi Obi Low

### Population structure

**Figure 2:**
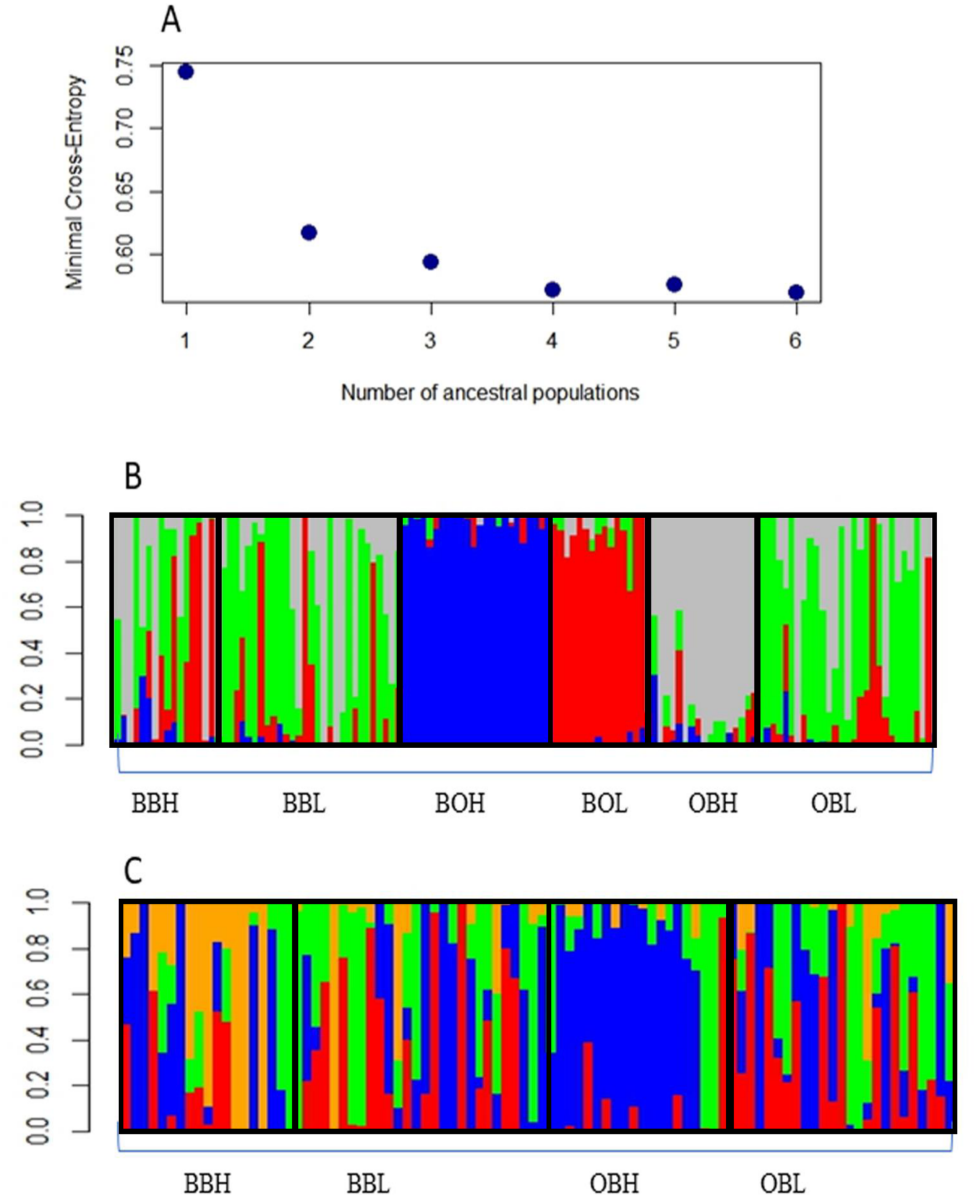
(A) Minimal Cross-entropy and ancestral population. A plateau is shown cluster 4, so the best number of clusters for this data set is 4. (B) Number of clusters for six sites for *K* = 4. The four clusters are: Boolumba High (BOH), blue; Booloumba Low (BOL), red; Obi Obi High (OBH), grey; and the rest is admixed. (C) Clusters without Booloumba sites, for *K* = 4, there are 2 clusters, Only Obi Obi High (OBH), blue came out as a separate cluster and the rest are admixed.

Admixture analysis with the R package LEA indicated that the most likely number of clusters was 4, as the graph reached a plateau after 4 clusters (Fig. 2A). Figure 2B shows that 3 sample populations (BOH, BOL and OBH) are genetically distinct, as their individuals are mostly assigned to single, different genetic clusters. The remaining sample populations contain individuals that are an admixture of 3 or more genetic clusters. Figure 2C shows that only one sample population (OBH) has individuals that are mostly allocated to one genetic cluster.

**Figure 3:**
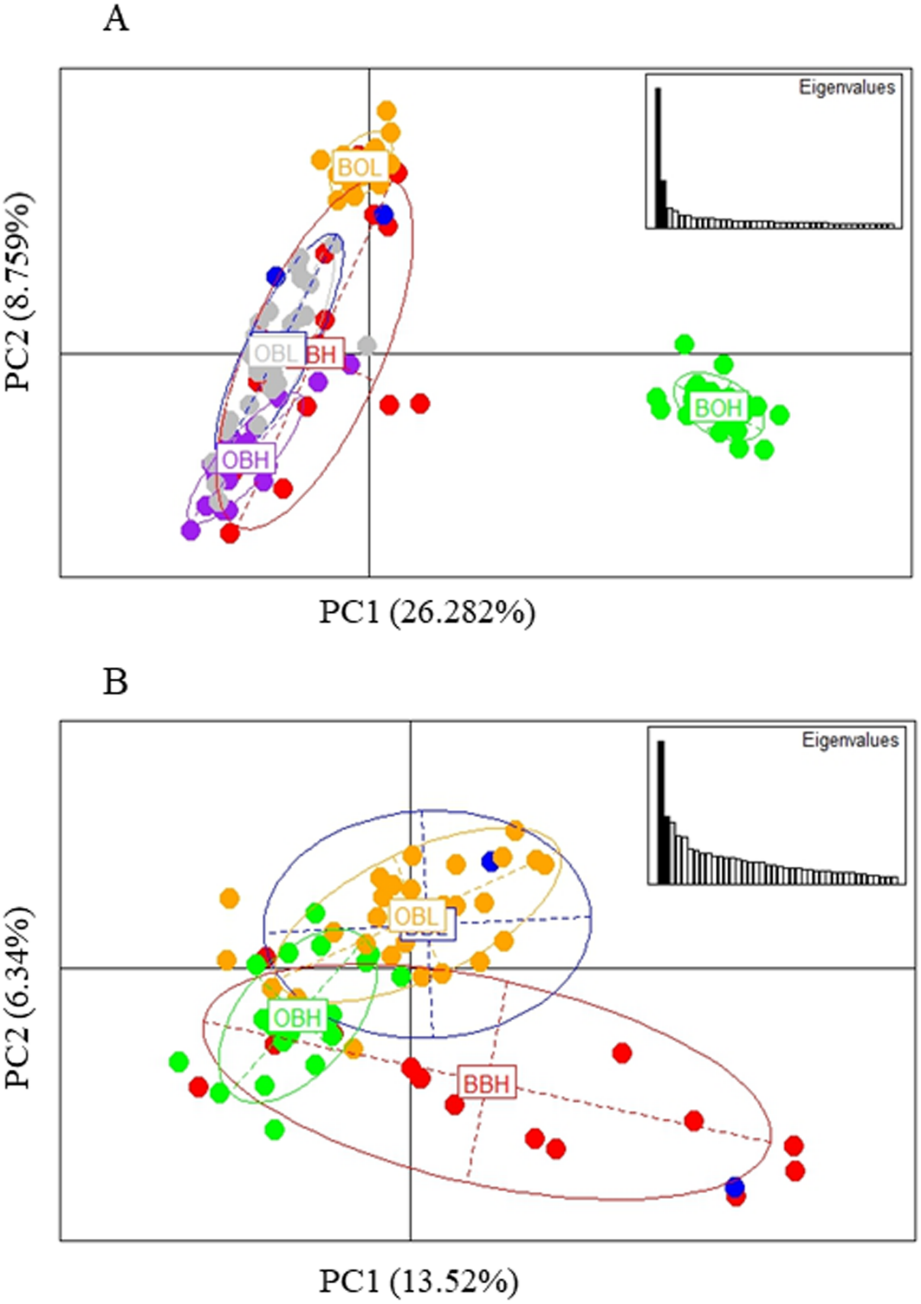
(A) PCA of 6 sites based on PC1 and PC2 (Broken Bridge High, BBH; Broken Bridge Low, BBL; Boolumba High, BOH; Booloumba Low, BBL; Obi-Obi High, OBH; Obi-Obi Low, OBL) Boolouma High population is clearly differentiated from the rest. (B) PCA of four sites excluding Booloumba sites (Broken Bridge High, BBH; Broken Bridge Low, BBL; Obi-Obi High, OBH; Obi-Obi Low, OBL).

The PCA based method of population clustering showed clear separation of BOH from all other sites based on Principal Component one (PC1) which accounted for 26.28% of the variance (Fig. 3A). Since Booloumba Creek populations were so divergent from the other sites, PCA analysis was repeated without Booloumba populations, but it still identified no clear grouping between the other 4 populations (Fig. 3B). PCA was also performed excluding loci putatively under selection (27 outliers) but the population structure was similar to the structure including all loci (Fig. S1).

In summary, all three methods suggested that the two Booloumba populations were the most different and different from each other. OBH was slightly differentiated from OBL, BBH and BBL by admixture analysis although this was less clear in the PCA.

### Detection of outliers

**Figure 4:**
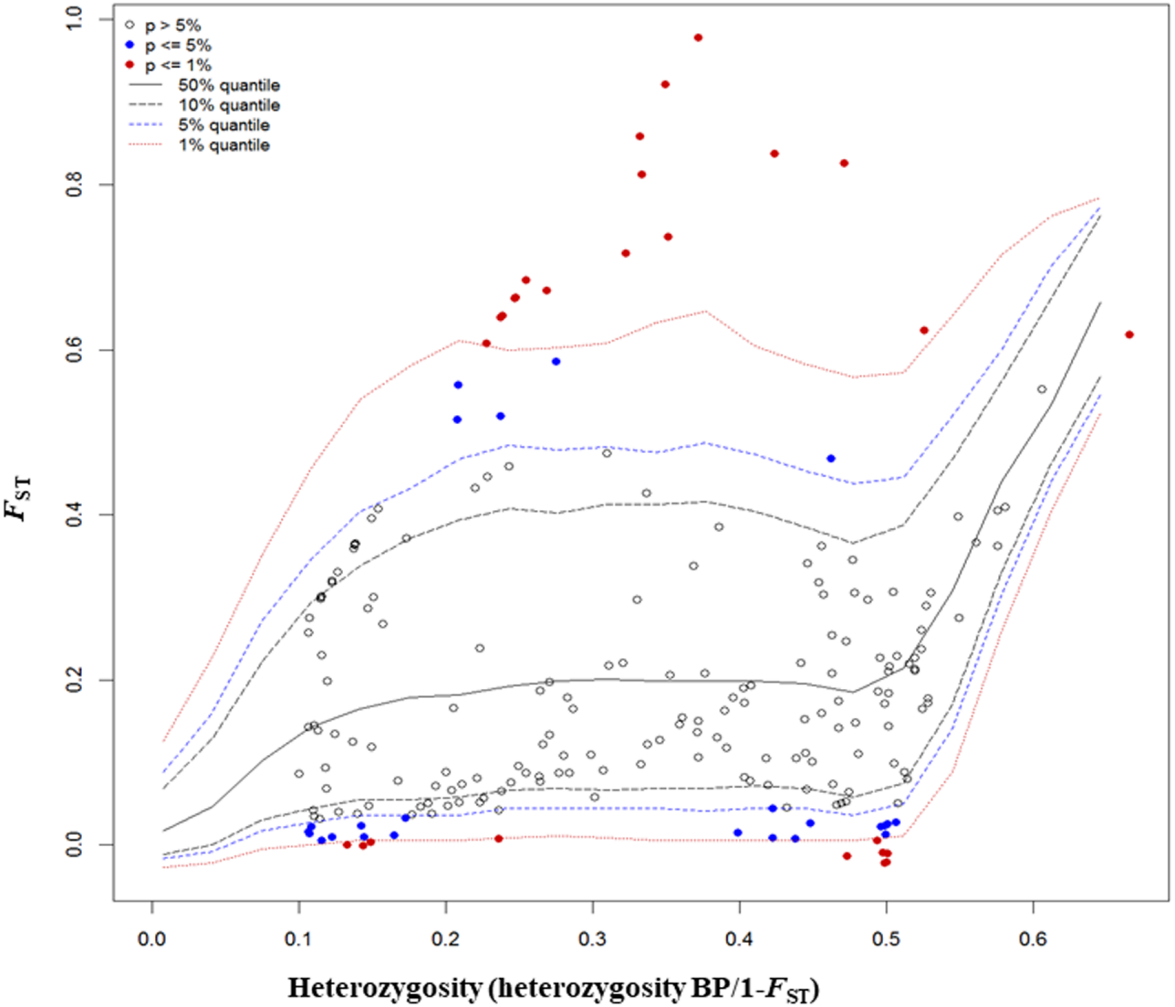
Results from *F*_*ST*_ outlier test in Arlequin including six sites using 213 SNPs and a hierarchical island model implemented in Arlequin. Three types of loci are observed: loci that fall between 95% quantiles are neutral loci indicated as white circles, loci that fall below the 1% quantile are under balancing selection indicated by red dots down the bottom of the plot, and loci that fall above the 99% quantile are under divergent selection, indicated by red dots on the top.

The *F*_ST_ outlier test in Arlequin produced 27 outliers (12.67%) at the 0.1% level of significance and 186 loci (87.32%) were neutral (Fig. 4). Among these 27 loci, 12 loci were identified as being as under balancing selection while 15 loci were under divergent selection.

In Bayescan using prior odds of 10, only 1 locus was identified as an outlier (locus 90) at FDR of 0.001 and was potentially under divergent selection with a posterior probability of 1 (PO).

The PCA based method, detected only 1 outlier (locus 41) correlated with PC1, with a False Discovery Rate (*q* value) threshold of 0.001 (Table S2, Fig. S2).

**Figure 5:**
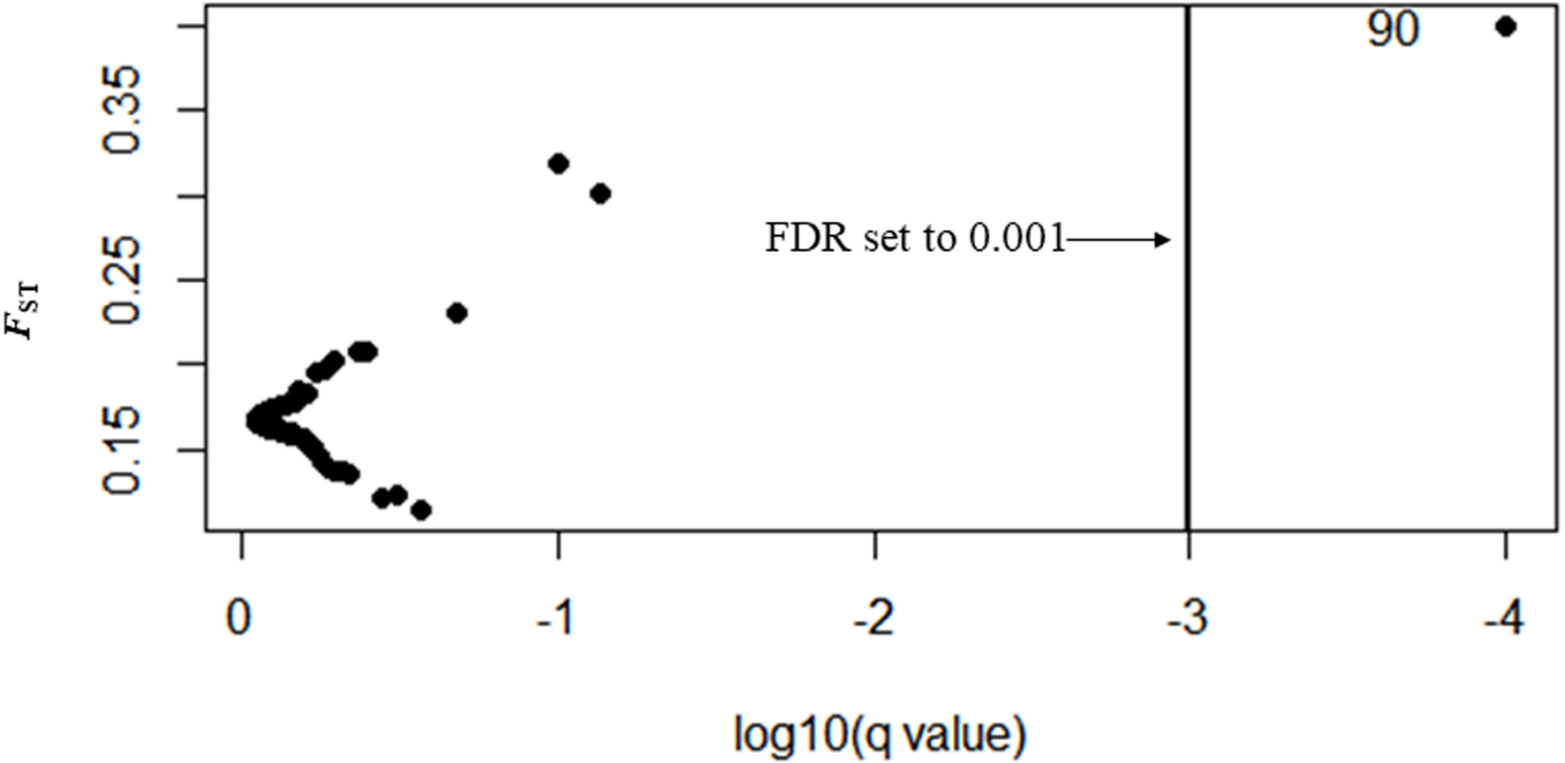
BAYESCAN plot using 213 SNPs including six sites of *Paratya australiensis*. *q*-value which is analogous to FDR has been set to 0.001 (if *q* = 0.001, log10q = −3). This is indicated with a vertical line. Loci which fall to the right of the line are under selection, locus ID 90 is putatively under selection.

In total, 27 outliers were identified from any of the three methods (*F*_ST_ outlier, Bayesian and PCA based method). Locus number 90 was identified in *F*_ST_ outlier and Bayesian methods (Fig. 5). In the *F*_ST_ outlier method, locus 90 came out with a very high *F*_ST_ value, in Bayesian it was in decisive selection (High).

Locus number 41 was identified as an outlier by both the *F*_ST_ outlier method and the PCA based method. The *F*_ST_ outlier method identified that it was under divergent selection and in PCA based method it was correlated with PC1.

### The outliers

Based on the genotype frequency of the 27 outliers, there were fixed differences at several loci between Booloumba Creek populations. Some loci showed significant deficiency or excess of heterozygotes compared to HWE expectations. Among them, 1 locus in BBL had fewer heterozygotes than expected under HWE. One locus in BBH, 4 loci in BBL, 4 loci in BOH, 4 loci in OBL had more heterozygotes than expected under HWE. There were no loci in BOL or OBH that had excess or deficit in heterozygotes (Table S3).

### Parallel patterns of adaptation

### Matching outliers.

In Broken-Bridge Creek, of 1,716 loci, 14 loci were identified as possibly under divergent selection, falling above the 99% quantile in the *F*_ST_ outlier test in Arlequin. There were no loci identified as under balancing selection (below 1% quantile). The Bayesian method did not detect any outliers for Broken Bridge Creek using prior odds of 100. In contrast, the PCA based method revealed 159 outliers (9.27%) out of 1716 loci and outliers were correlated with PC1 and PC2. 9 loci were common to both the *F*_ST_ outlier method and PCA based method.

In Booloumba Creek, out of 704 loci, 22 were identified as possibly under divergent selection and there were no loci inferred to be under balancing selection with the *F*_ST_ outlier method in Arlequin. In the PCA based method among 704 loci, 16 loci were outliers (2.27%) correlated with Principal components 1 and 2. 6 loci were common to both the *F*_ST_ outlier and the PCA based methods. However, no outliers were detected using the Bayesian method.

In Obi Obi Creek out of 499 loci, 4 loci (0.8%) were identified as outliers under divergent selection and there were no loci under balancing selection. The Bayesian method did not detect any outliers between high elevation and low elevation populations of Obi Obi Creek. The PCA based method detected 5 outliers that (1.00%) were related to PC1 and PC2. No outlier was common to the *F*_ST_ outlier and the PCA based method.

The outliers detected in each stream using the 3 different methods were matched (catalog ID) against each other to detect a possible parallel pattern of adaptation. There were no matches between any two streams.

### Parallel patterns based on PCA.

**Figure 6:**
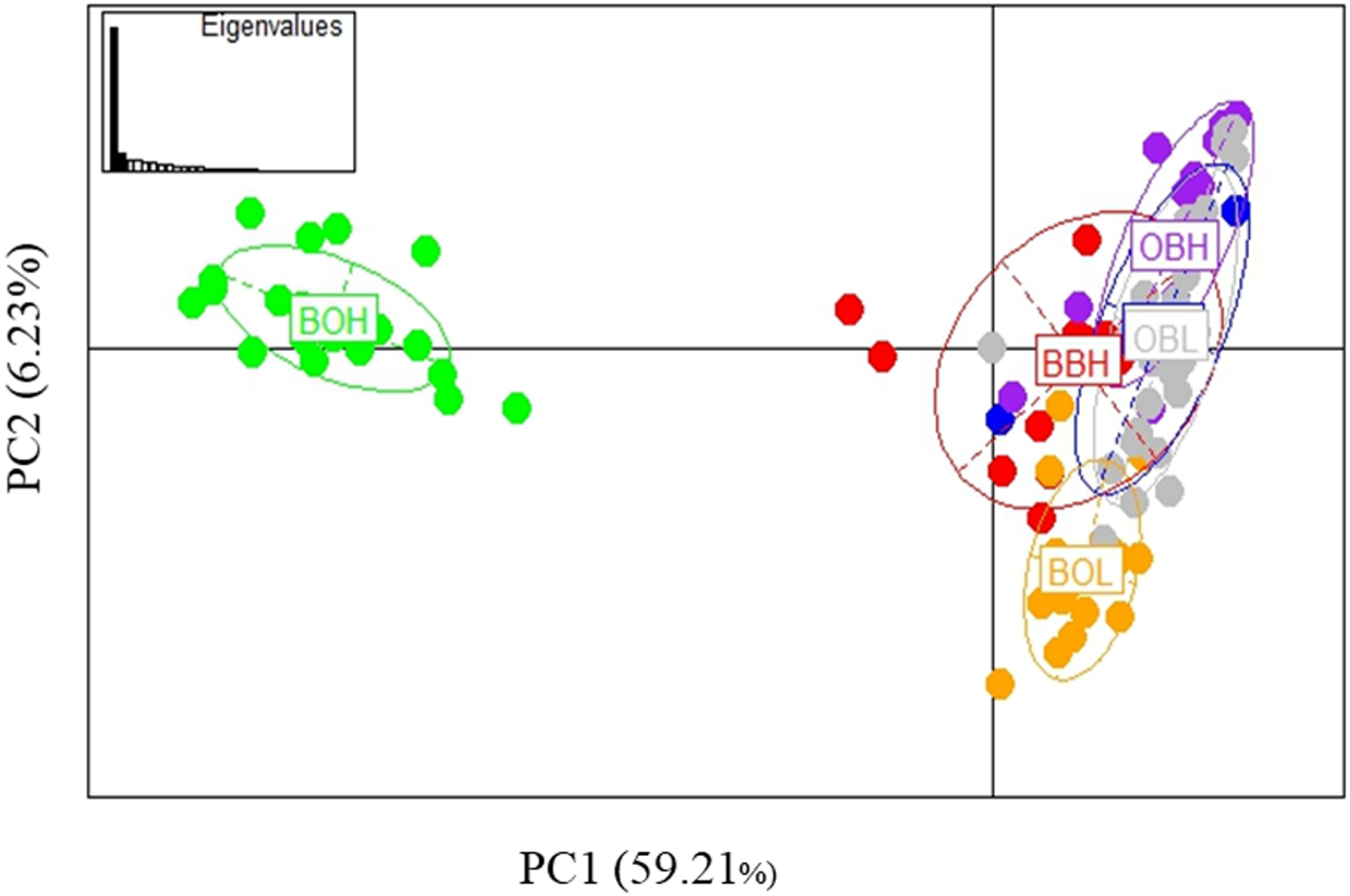
PCA based on 27 outlier loci. Analysis done on six sites including 131 individuals.

Possible parallel patterns of adaptation were examined based on the 27 outliers (all 6 populations) identified from all three methods (Fig. 6), but there was no clear pattern of parallel adaptation. If there were parallel patterns, all upstream populations would group together, and all downstream populations would group together. However, this condition would only be visible if the selection pressure is similar in all 3 localities. Only the BOH population was separated from the BOL indicating genetic differentiation, but this pattern was not shown in the other two stream populations. Similar to the analysis in Fig. 3A including all loci, the PCA based on outliers also showed that only Booloumba High population was very distinct from the rest (Fig. 6). The only difference was that the variance based on outliers (PC1-59.21%) was much higher than the neutral (PC1-20.995%) comparison (Fig. S1).

### BLAST

BLAST (NCBI) of the outlier loci (27 outliers) did not show any very similar sequence match (megablast) with the *Macrobrachium rosenbergii* sequence database. However, with BLASTn (somewhat similar sequence) most loci showed a small (10–36%) sequence match (Table S4).

### mtDNA sequencing

Individuals from BOH and BOL populations aligned with Reference lineage 4 sequence in the phylogenetic tree (Fig. S3). Due to strong structuring it was thought that BOH and BOL populations may have belonged to different lineages, but results showed that they both belonged to lineage 4.

## Discussion

The overall aim of the study was to identify population structure between high and low elevation populations using a genomic approach. Furthermore, loci, putatively under selection were also determined to indicate any evidence of adaptation to altitude.

### Population divergence

The Booloumba Creek High (BOH) population had a number of private alleles, which reflects the isolation of the high elevation population from the low elevation population and suggests extremely limited dispersal. In contrast to many other studies, higher genetic diversity was observed in BOH than BOL. Lower diversity has been reported at high elevation sites for *Gambusia holbrooki* (Congdon, 1995) and also in *Paratya australiensis* (Hughes et al., 1995). The result for Booloumba Creek is surprising because the potential for high dispersal to high elevation is likely to be low as there is a >10 m waterfall separating the two sites. According to Bunn & Hughes (1997) instream movement of invertebrates in the Conondale range has been very limited due to the physical nature of the streams which create barriers for dispersal.

The *F*_ST_ value (0.446) between the high altitude and low altitude Booloumba populations was very large, indicating restricted gene flow and limited dispersal between them. In contrast, Broken Bridge (*F*_ST_ = 0.04289) and Obi Obi Creek (*F*_ST_ = 0.00737) showed comparatively more gene flow between high and low altitudes, as the genetic differentiation was low. The overall pattern of population structure is concordant with past findings based on a few allozyme loci, which revealed that among the 4 sub-catchments, Booloumba and Kilcoy sub catchments showed the highest genetic differentiation and Mary (including Broken Bridge Creek) and Stony sub-catchments showed least genetic differentiation (Hughes et al., 1995). An explanation for such genetic differentiation could be the fact that shrimps from high elevation are washed down as larvae, but they do not survive due to different environmental conditions (eg higher temperature) at low elevation. This would need to be investigated further.

In 1995, Hughes et al. reported a mean *F*_ST_ value of 0.378 among Booloumba Creek sampled populations, which is remarkably similar to that reported here. This result tends to suggest that conclusions from early allozyme studies may be just as reliable as current ones using thousands of SNP’s.

The strong genetic structure is supported further by PCA and admixture analyses. The situation in Booloumba Creek could be explained by the theory of Lowe et al. (2006). According to Lowe et al. (2006) “isolation by slope” is sometimes responsible for population divergence where populations are isolated by increasing altitudinal difference. They also suggest that steeper gradients of the headwater section play a significant role in population differentiation and restricted dispersal. Cook, Bunn & Hughes (2007) also suggested altitudinal gradient to be a negative factor for *Paratya australiensis* dispersal in the Granite Creeks area in south-east Australia. However, there was no significant genetic structure in the other two creeks which could be due to ongoing geneflow between high elevation and low elevation populations. On the other hand, in Booloumba Creek there might have been chance dispersal to new habitat followed by local adaptation and complete isolation. Isolation of these populations for extended period lead to further divergence due to genetic drift.

Such strong structure could be explained by the presence of different lineages (or cryptic species) occur in the two BOH and BOL populations. However, the phylogenetic analysis based on a fragment of the mitochondrial COI gene showed that the individuals from high and low elevation populations were from the same lineage (4) (Rahman, Schmidt & Hughes, 2018).

Highly structured populations have been identified in other stream species, for example the purple spotted gudgeon- *Mogurnda adspersa* (Hughes et al., 2012), in guppies- *Poecilia reticulata* (Barson, Cable & Van Oosterhout, 2009) and in Caridina-*Caridina zebra* (Hughes et al., 1996). This has been suggested to result from strong genetic drift in small, isolated populations (Barson, Cable & Van Oosterhout, 2009). Other examples include, flyspecked hardy heads (*Craterocephalus stercusmuscarum*) in north Queensland, where high and low altitude populations show high genetic differentiation that was suggested to have resulted from different historical colonization events (McGlashan & Hughes, 2000).

It is likely that a long period of isolation has allowed some degree of adaptation in Booloumba populations. The high elevation populations may be adapted to the cooler environment of the high elevation site (temperature difference of 1−2 °C) while the low elevation populations may be adapted to the slightly warmer environment as was suggested by Fawcett, Hurwood & Hughes (2010), in a study of *P*. *australiensis* populations on the other side of the Conondale Range. Furthermore, ∼3% of loci were identified by the *F*_*ST*_ test as being outliers in Booloumba Creek, compared to ∼1% and 0.8% in the other two creeks.

### Putative genomic region under selection

Outlier analysis identified putative loci under selection. Detailed analysis of the outliers showed that there was fixation of alternate alleles in high elevation and low elevation populations for a few loci, but only in Booloumba Creek. Also, some loci showed frequency differences, which could be expected if selection was influencing them but was not strong enough to counteract other processes, such as geneflow. Booloumba Creek populations showed the most differences between high elevation and low elevation but distinguishing the effect of isolation (lack of gene flow) from selection is difficult. High elevation and low elevation populations in Booloumba are very different and have probably been isolated for a considerable time. This conclusion is also supported by earlier allozyme studies (Hughes et al., 1995) and our analysis of neutral alleles. Therefore, if favorable alleles were present in either upstream or downstream locations, they might be able to persist for longer and become fixed if there has been no gene flow for long periods. On the other hand, there was little evidence of fixation of alleles in Broken Bridge or Obi Obi Creek populations. In the single stream analysis, outliers were identified in each stream, suggesting points in the genome that resist the homogenizing influence of gene flow and could reflect the effects of selection.

Details on the outliers showed that there were a few loci that had deficiency/excess of heterozygote genotypes (than expected under HWE) in BBH, BBL, BOH and OBL populations. Few of these loci were common across these 4 populations. The percentage of outliers detected in this study was 12.67% according to the *F*_ST_ outlier method, which is high compared to the percentage of outliers expected (5–10%) suggested by Nosil, Funk & Ortiz-Barrientos (2009). Some other studies also reported outliers at lower percentages within a species, for example 6.7% of outliers in Pacific Salmon (Larson, 2015) and 3.5–6.5% in wild guppies (Willing et al., 2010). However, when two species of manakins were compared a large percentage of outliers (25.2%) was detected (Parchman et al., 2013). Narum et al. (2010) studied montane and desert populations of redband trout (*Oncorhynchus mykiss gairdneri*) and they found 7.89% loci under selection. They concluded that with outlier analysis it is possible to identify putative genomic regions under selection but without pre-information about the species genome, it is difficult to determine specific loci/genes causing adaptation to different environmental conditions (desert/montane). Adaptation studies in natural populations have always taken into consideration the environmental conditions in which the organism resides because genetic variance is strongly related to the environment in which it is measured. Comparing populations from different environments indicates strong interaction between the expression of genetic variance and environmental difference (Ellegren & Sheldon, 2008). As outlier loci may indicate the degree of adaptive divergence among populations, the percentage of outliers can depend on the difference in environmental conditions of the populations concerned (Luikart et al., 2003).

Despite the fact that outliers were identified in the study from high and low elevation stream populations of *Paratya australiensis*, these are not necessarily candidate loci for adaptation. It was not possible to test this because there was no *Paratya* reference genome with which to compare the outliers. It is believed that outliers may lie in the genomic regions showing very high levels of genetic differentiation (Nosil, Funk & Ortiz-Barrientos, 2009). However, candidate loci may be missed using RAD-seq technique, or they may be closely linked to the actual loci under selection.

When outlier loci were BLASTed against the *Marobrachium rosenbergii* sequence database in GenBank, there was no match with any known functional genes. It is suspected that *Paratya australiensis* has a large genome and thus markers produced by RAD-seq produces markers are likely to screen only a small portion of the genome, so this process might have missed regions under selection.

There are other factors that could have influenced the results. (1) environmental conditions between populations were not massively different. The detection of outliers may require very strong environmental differences between populations. In our study, high elevation and low elevation populations in Booloumba Creek differed markedly in altitude while the other two creeks were less different. Hence, if these populations are separated for long periods of time, selection and drift will act on the population in a manner that makes it impossible to differentiate between them. Thus, loci under selection have the same population genetic structure as those evolving via drift (2) Low number of loci that matched across individuals. Most probably, RAD libraries did not have enough breadth of coverage which could be either due to large genome size or other technical issues. If a large number of loci (thousands) were matched evenly across individuals, we would have had a better chance of detecting potential outliers under selection. It is quite possible that the outliers identified in the present study are false positives, because in a fractal landscape like the stream environment, population genetic parameters are correlated with the number of demes. Hence, the variance of *F*_ST_s are influenced by the fractal geometry resulting in a higher rate of outliers that are false positives (Fourcade et al., 2013). According to Fourcade et al. (2013) a simulation on networks can be used to calculate the actual variance of *F*_ST_. However, in the present study we did not use simulation but used three different methods for calculating outliers. So, we recommend that simulation should be used for future studies. 3) Sequencing depth/coverage may not have been sufficient. This is also unlikely here, as 83% of individuals had a good depth of coverage (average 19.4x). In relation to our assumption at the beginning of the study, we are unable to distinguish between drift vs selection as we observed a similar pattern of genetic structure using outliers and neutral loci. So, in this scenario RAD-seq data could not differentiate the two processes and an additional RNA-seq analysis would be beneficial to describe the selection process better.

According to Slatkin (1985) allele frequencies will tend to diverge due to genetic drift or natural selection among populations in the presence of limited dispersal. So, divergent selection might be an explanation for the population divergence in Booloumba Creek. Broken Bridge and Obi Obi Creek did not show strong divergence and population structuring, although presence of outliers in individual stream analysis gave some evidence of selection. Wit & Palumbi (2013) studied transcriptomes of 39 red abalone (*Heliotis rufescens*) from three regions along the US west coast that differ in a number of environmental parameters (temperature, aragonite saturation, exposure to hypoxia and disease pressure). Population structuring with PCA using 21,579 SNPs showed no well-defined structure among the three regions (Monterey Bay, Sonoma and Cape Mendocino) although 691 SNP showed significantly higher genetic differentiation than expected in an *F*_ST_-outlier analysis (Wit & Palumbi, 2013). So, despite the fact that there was no clear grouping of populations from different environments, they concluded that there was a signature of selection (Wit & Palumbi, 2013).

This study was designed to detect differences between altitudes, so the presence of outliers was to indicate adaptation to different altitudes. However, in order to relate the adaptive loci to water temperature or other environmental factors, further studies would be required. Studies on other organisms have shown adaptation and allele frequency difference along altitudinal gradients e.g., in common frog in North French Alps (Bonin et al., 2005), in small rodents in Swiss Alps (Fischer et al., 2011), in European trout population (Keller, Taverna & Seehausen, 2011) and in Japanese conifers (Tsumura et al., 2012). In comparison to these studies where altitudinal differences were very large (∼2000 m asl), the current study dealt with relatively small differences in altitude ∼400 m asl with a difference in water temperature from 1−2 °C. However, temperature could be one of the drivers of local adaptation of *P*. *australiensis* in this case. Several studies indicated that environmental factors may limit gene flow and cause population divergence due to selection in local environments (Narum et al., 2008; Narum et al., 2010).

### Population grouping and adaptation

The PCA with only neutral loci showed a similar pattern to PCA with outliers and PCA with all loci included. This could imply that adaptive divergence was not strong enough to influence overall population divergence as adaptive and neutral population divergence were similar. If the outliers represented loci under strong selection, genetic divergence based on neutral loci only would be lower, depending on when the initial population divergence occurred. The major divergence was in Booloumba Creek, which may have affected the overall analysis in the present study. Keller, Taverna & Seehausen (2011) observed European trout populations in major drainage systems (Rhone and Pho drainage system in Europe) and found higher divergence within drainages and geographic proximity influenced neutral divergence. Furthermore, Keller, Taverna & Seehausen (2011) mentioned that between drainage, divergence was higher based on outlier loci. However, in our study there was no difference between adaptive (based on outlier loci) and neutral divergence (based on neutral loci only). If the adaptive divergence was stronger, populations would be expected to show more divergence based on outliers than on neutral loci only. Again, this is dependent on the amount of time populations have been isolated.

### Parallel patterns of adaptation

The number of outliers common to the *F*_ST_ outlier method and the PCA based method were: 9 loci in Broken Bridge Creek, 6 loci in Booloumba Creek and zero in Obi Obi Creek. As the PCA was done based on the outliers only and showed divergence of populations (especially Booloumba populations) we could infer that the divergence was due to some degree of adaptation and there was adaptive divergence in Booloumba populations. However, there were no matching loci across any streams. According to Keller, Taverna & Seehausen (2011), the same loci may not behave as outliers in all drainages and this is due to the fact that the amount and type of adaptive genetic variation varies between drainages. Phillips et al. (2016) looked for a genome wide hard selective sweep in *Drosophila melanogaster*. They did not find any candidate alleles that were fixed in different populations but they identified genomic regions showing similar genetic variation across independent replicate populations indicating a parallel pattern of selection.

Twenty-seven (27) outliers identified in the overall analysis were used to test parallel patterns based on PCA. In parallel patterns of local adaptation, populations inhabiting the same environmental conditions or from contrasting situations should show a similar pattern of groupings (Kawecki & Ebert, 2004). Based on the PCA, there was no parallel pattern as the populations did not group according to up and downstream environments across all streams. The only grouping was observed in Booloumba Creek. Neither matching loci, nor the PCA showed parallel pattern of adaptation in this case.

Similar results were observed in two male color types of Lake Victoria Cichlid using 107 SNP markers (Keller et al., 2013). Also, a parallel pattern of adaptation was studied in Sockeye Salmon from Wood River and Lake Iliamna in the North-western Bristol Bay, USA using a set of known adaptive markers for pairs of populations from different ecotypes (beach and other). PCoA showed that beach populations from both Wood River and Iliamna Lake grouped together indicating a parallel pattern of adaptation (Larson, 2015). In our study there was no parallel pattern, with the evidence not strong enough to come to a conclusion about parallel patterns of adaptation.

## Conclusion

In conclusion, this study used genomic approaches to observe population structure and local adaptation in the Australian glass shrimp (*P*. *australiensis*). In this study we evaluated evidence of local adaptation. We were interested to observe if divergence among populations is influenced more by selection or genetic drift in this part of the Conondale Range. Sites were selected based on altitudinal differences that differed in water temperature difference by 1−2 °C. We found similar genetic structure for both neutral and putative loci under selection inferring that there is no clear indication that the divergence is due to drift or selection. However, drift and selection are not mutually exclusive processes, so divergence could have been caused by selection initially but could show similar pattern to genetic drift if dispersal has been limited in the system. For the Booloumba Creek populations, there might have been chance dispersal to new habitat followed by local adaptation and complete isolation from the parental population, leading to further divergence due to genetic drift.

##  Supplemental Information

10.7717/peerj.8139/supp-1 Figure S1 PCA based on 186 neutral loci Analysis done on 6 populations including 131 individuals.

10.7717/peerj.8139/supp-2 Figure S2 PCA of the 6 populations (213 SNP) using Pcadapt package BOH population was separate based on PC1.

10.7717/peerj.8139/supp-3 Figure S3 Bayesian tree of Booloumba Creek individuals along with reference sequences Numbers on branches refers posteriors.

10.7717/peerj.8139/supp-4 Table S1 List of Sample ID from different populations

10.7717/peerj.8139/supp-5 Table S2 Outliers detected based on PCA method (PCadapt)

10.7717/peerj.8139/supp-6 Table S3 Genotype frequency of the outliers for each population Black = common alleles, White = rare alleles and Grey = heterozygotes. “+” indicates presence of fewer heterozygotes than expected under HWE and “-” indicates more heterozygotes than expected under HWE.

10.7717/peerj.8139/supp-7 Table S4 Showing BLASTn results of the 27 outliers matched against *Macrobrachium rosenbergii* sequence database

10.7717/peerj.8139/supp-8 Data S1 Genetic structure Raw Data

## Additional Information and Declarations

### Data Availability

The following information was supplied regarding data availability: Dryad
