# Supplementary figures and images for: Genetic structure of Australian glass shrimp, Paratya australiensis, in relation to altitude
Source: PeerJ. 2020 Jan 9;8:e8139. doi: 10.7717/peerj.8139 (PMC6955102; doi:10.7717/peerj.8139)

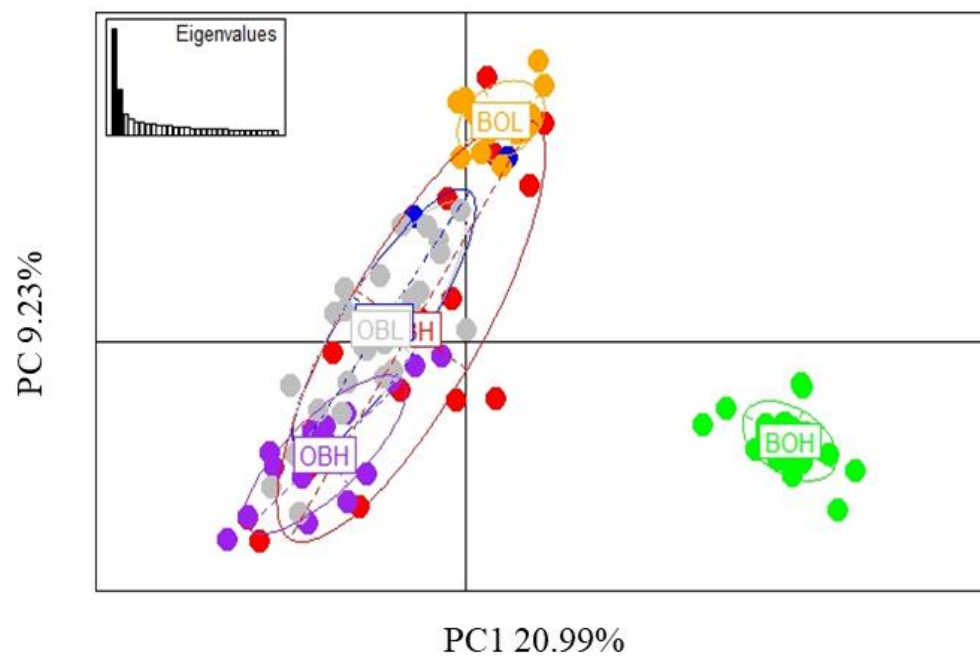

Supplement: Figure S1 — Analysis done on 6 populations including 131 individuals. [file peerj-08-8139-s001.pdf]

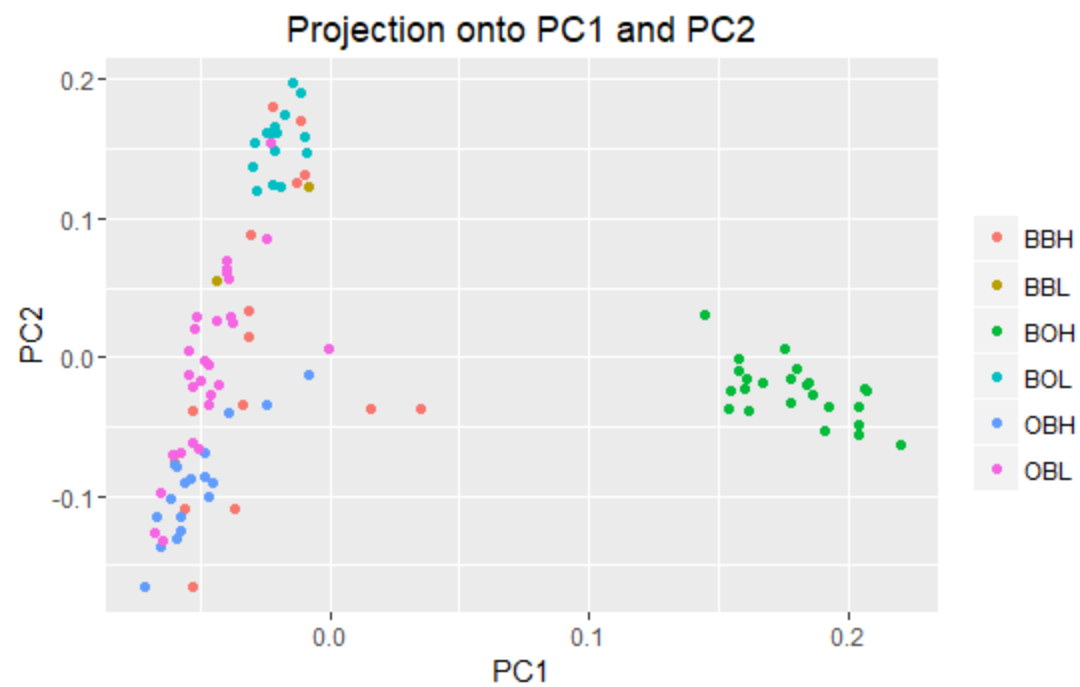

Supplement: Figure S2 — BOH population was separate based on PC1. [file peerj-08-8139-s002.pdf]

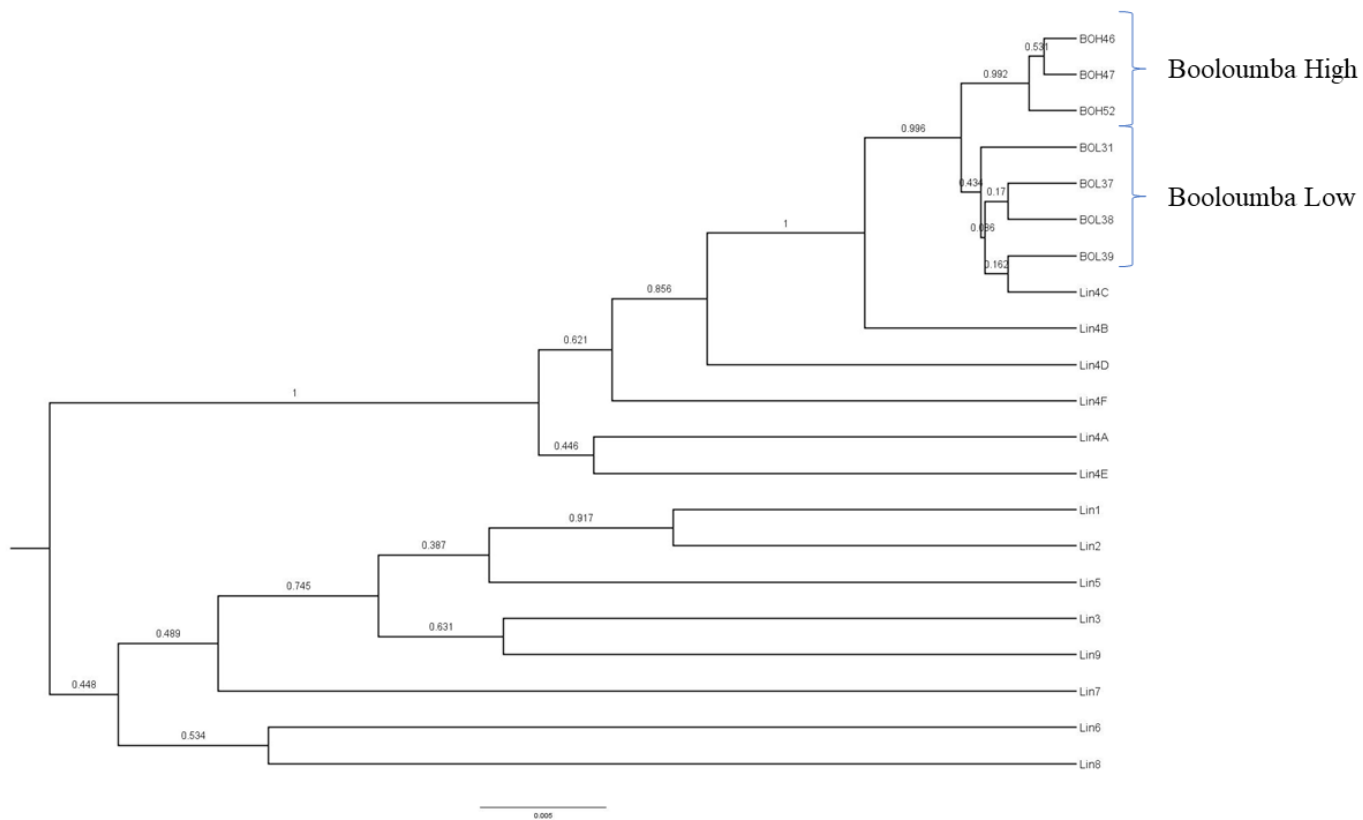

Supplement: Figure S3 — Numbers on branches refers posteriors. [file peerj-08-8139-s003.pdf]
