# Supplementary material for: Genetic structure of Australian glass shrimp, Paratya australiensis, in relation to altitude
Source: PeerJ. 2020 Jan 9;8:e8139. doi: 10.7717/peerj.8139 (PMC6955102; doi:10.7717/peerj.8139)
Supplement: Table S1 [file peerj-08-8139-s004.docx]

Table S1. List of Sample ID from different populations

| **Stream Name** | **Population Name** | **Sample ID** |
| --- | --- | --- |
| **Broken Bridge Creek** | **Broken Bridge High (BBH)** | BBH121 |
|  |  | BBH122 |
|  |  | BBH124 |
|  |  | BBH130 |
|  |  | BBH94 |
|  |  | BBH95 |
|  |  | BBH98 |
|  |  | BBH99 |
|  |  | BBH104 |
|  |  | BBH105 |
|  |  | BBH106 |
|  |  | BBH108 |
|  |  | BBH110 |
|  |  | BBH134 |
|  |  | BBH135 |
|  |  | BBH137 |
|  |  | BBH144 |
|  |  | BBH149 |
|  |  | BBH85 |
|  | **Broken Bridge Low (BBL)** | BBL215 |
|  |  | BBL238 |
|  |  | BBL250 |
|  |  | BBL218 |
|  |  | BBL239 |
|  |  | BBL251 |
|  |  | BBL152 |
|  |  | BBL220 |
|  |  | BBL244 |
|  |  | BBL252 |
|  |  | BBL160 |
|  |  | BBL224 |
|  |  | BBL245 |
|  |  | BBL253 |
|  |  | BBL203 |
|  |  | BBL230 |
|  |  | BBL246 |
|  |  | BBL254 |
|  |  | BBL208 |
|  |  | BBL233 |
|  |  | BBL247 |
|  |  | BBL255 |
|  |  | BBL212 |

Table S1. List of Sample ID from different populations (Cont.)

| **Stream Name** | **Population Name** | **Sample ID** |
| --- | --- | --- |
| **Broken Bridge Creek** | **Broken Bridge Low (BBL)** | BBL234 |
|  |  | BBL248 |
|  |  | BBL256 |
|  |  | BBL184 |
|  |  | BBL236 |
|  |  | BBL249 |
| **Booloumba Creek** | **Booloumba High (BOH)** | BOH11 |
|  |  | BOH13 |
|  |  | BOH15 |
|  |  | BOH19 |
|  |  | BOH20 |
|  |  | BOH24 |
|  |  | BOH34 |
|  |  | BOH35 |
|  |  | BOH36 |
|  |  | BOH53 |
|  |  | BOH59 |
|  |  | BOH62 |
|  |  | BOH64 |
|  |  | BOH67 |
|  |  | BOH69 |
|  |  | BOH75 |
|  |  | BOH77 |
|  |  | BOH79 |
|  |  | BOH82 |
|  |  | BOH83 |
|  |  | BOH84 |
|  |  | BOH85 |
|  |  | BOH86 |
|  |  | BOH87 |
|  | **Booloumba Low (BOL)** | BOL11 |
|  |  | BOL15 |
|  |  | BOL17 |
|  |  | BOL30 |
|  |  | BOL54 |
|  |  | BOL89 |
|  |  | BOL101 |
|  |  | BOL110 |
|  |  | BOL111 |
|  |  | BOL112 |
|  |  | BOL113 |
|  |  | BOL114 |

Table S1: List of Sample ID from different populations (Cont.)

| **Stream Name** | **Population Name** | **Sample ID** |
| --- | --- | --- |
| **Booloumba Creek** | **Booloumba Low (BOL)** | BOL115 |
|  |  | BOL116 |
|  |  | BOL119 |
|  |  | BOL120 |
|  |  | BOL121 |
|  |  | BOL123 |
|  |  | BOL125 |
|  |  | BOL126 |
|  |  | BOL127 |
|  |  | BOL128 |
|  |  | BOL129 |
| **Obi Obi Creek** | **Obi Obi High (OBH)** | OBH140 |
|  |  | OBH142 |
|  |  | OBH177 |
|  |  | OBH178 |
|  |  | OBH179 |
|  |  | OBH180 |
|  |  | OBH181 |
|  |  | OBH182 |
|  |  | OBH183 |
|  |  | OBH184 |
|  |  | OBH185 |
|  |  | OBH186 |
|  |  | OBH187 |
|  |  | OBH188 |
|  |  | OBH189 |
|  |  | OBH190 |
|  |  | OBH191 |
|  |  | OBH192 |
|  |  | OBH193 |
|  | **Obi Obi Low (OBL)** | OBL1 |
|  |  | 2 |
|  |  | 3 |
|  |  | 4 |
|  |  | 5 |
|  |  | 6 |
|  |  | 7 |
|  |  | 8 |
|  |  | 9 |
|  |  | 11 |

Table S1: List of Sample ID from different populations (Cont.)

| **Stream Name** | **Population Name** | **Sample ID** |
| --- | --- | --- |
| **Obi Obi Creek** | **Obi Obi Low** | OBL12 |
|  |  | OBL13 |
|  |  | OBL14 |
|  |  | OBL15 |
|  |  | OBL16 |
|  |  | OBL17 |
|  |  | OBL19 |
|  |  | OBL20 |
|  |  | OBL21 |
|  |  | OBL22 |
|  |  | OBL23 |
|  |  | OBL24 |
|  |  | OBL25 |
|  |  | OBL26 |
|  |  | OBL27 |
|  |  | OBL29 |
|  |  | OBL30 |
