# Supplementary material for: Genetic structure of Australian glass shrimp, Paratya australiensis, in relation to altitude
Source: PeerJ. 2020 Jan 9;8:e8139. doi: 10.7717/peerj.8139 (PMC6955102; doi:10.7717/peerj.8139)
Supplement: Table S2 [file peerj-08-8139-s005.docx]

Table S2. Outliers detected based on PCA method (PCadapt)

| Serial No | Locus Id | PC |
| --- | --- | --- |
| 1 | 4 | 7 |
| 2 | 12 | 7 |
| 3 | 18 | 6 |
| 4 | 41 | 1 |
| 5 | 55 | 7 |
| 6 | 100 | 6 |
| 7 | 110 | 4 |
| 8 | 114 | 6 |
| 9 | 119 | 6 |
| 10 | 131 | 8 |
| 11 | 133 | 6 |
| 12 | 192 | 3 |
