# Supplementary material for: Genetic structure of Australian glass shrimp, Paratya australiensis, in relation to altitude
Source: PeerJ. 2020 Jan 9;8:e8139. doi: 10.7717/peerj.8139 (PMC6955102; doi:10.7717/peerj.8139)
Supplement: Table S3 — Black = common alleles, White = rare alleles and Grey = heterozygotes. “+” indicates presence of fewer heterozygotes than expected under HWE and “-” indicates more heterozygotes than expected under HWE. [file peerj-08-8139-s006.docx]

| **Locus** | **BBH** | **BBL** | **BOH** | **BOL** | **OBH** | **OBL** |
| --- | --- | --- | --- | --- | --- | --- |
| 56352 |  | - | - |  |  | - |
| 85089 |  | + |  |  |  |  |
| 111763 |  | - | - |  |  | - |
| 138233 | - | - | - |  |  | - |
| 149036 |  | - | - |  |  | - |

**Table S3. Genotype frequency of the outliers for each population (Black=common alleles, White=rare alleles and Grey=heterozygotes. “+” indicates presence of fewer heterozygotes than expected under HWE and “-“ indicates more heterozygotes than expected under HWE).**
