# Supplementary material for: Genetic structure of Australian glass shrimp, Paratya australiensis, in relation to altitude
Source: PeerJ. 2020 Jan 9;8:e8139. doi: 10.7717/peerj.8139 (PMC6955102; doi:10.7717/peerj.8139)
Supplement: Table S4 [file peerj-08-8139-s007.docx]

Table S4. Showing BLASTn results of the 27 outliers matched against *Macrobrachium rosenbergii* sequence database

| Serial No | Outliers | Catalog ID | % Query cover *Macrobrachium* genus | Comment |
| --- | --- | --- | --- | --- |
| 1 | 41 | 32017 | 11% | D-loop partial sequence, mitochondrial |
| 2 | 90 | 64645 | 19% | Clone SBhMVJ2_3 microsatellite sequence |
| 3 | 152 | 99685 | 20% | D-loop partial sequence |
| 4 | 37 | 29800 | 12% | Ecdyson receptor (EcR-S2) mRNA |
| 5 | 146 | 96122 | No match | No match |
| 6 | 20 | 15987 | 16% | SUGbp8-124 microsatellite sequence |
| 7 | 195 | 138233 | 13% | Rab14mRNA, complete cds |
| 8 | 77 | 56352 | 13% | Rab5C mRNA, complete cds |
| 9 | 45 | 36513 | 23% | Complete mitochondrial genome |
| 10 | 118 | 81519 | 9% | Estrogen related receptor mRNA |
| 11 | 206 | 149036 | 9% | Microsatellite occ28-100 sequence |
| 12 | 161 | 111763 | 9% | COI gene, partial sequence |
| 13 | 67 | 49361 | 14% | Microsatellite Mro25 sequence |
| 14 | 179 | 126020 | 18% | Adipokinetic hormone related peptide precursor mRNA |
| 15 | 106 | 73017 | 28% | Clone SBhMVJ2 microsatellite sequence |
| 16 | 121 | 85089 | 9% | Clone SBhMT79112_7 microsatellite sequence |
| 17 | 36 | 29742 | 17% | Clone SUGbp12-3 microsatellite sequence |
| 18 | 126 | 87660 | 22% | Anti-lipopolysaccharide factor 2mRNA, partial cds |
| 19 | 122 | 85397 | 9% | Putative clotting protein mRNA, complete cds |
| 20 | 99 | 70333 | 11% | F male reproductive-related protein mRNA, complete cds |
| 21 | 213 | 151398 | 11% | Rab32 mRNA |
| 22 | 153 | 100595 | 14% | Mitochondrial complete genome |
| 23 | 21 | 16398 | 11% | Lectin 1 (Lec 1) mRNA |
| 24 | 82 | 59090 | No match | No match |
| 25 | 169 | 116708 | 13% | Clone SUGbp11-4 microsatellite sequence |
| 26 | 144 | 95527 | 9% | Transglutaminase mRNA, complete cds |
| 27 | **190** | **134038** | **36%** | mRNA for prophenoloxidase |
